# Multiparametric whole-body 3.0-T MRI in newly diagnosed intermediate- and high-risk prostate cancer: diagnostic accuracy and interobserver agreement for nodal and metastatic staging

**DOI:** 10.1007/s00330-018-5813-4

**Published:** 2018-12-05

**Authors:** Edward William Johnston, Arash Latifoltojar, Harbir Singh Sidhu, Navin Ramachandran, Magdalena Sokolska, Alan Bainbridge, Caroline Moore, Hashim Uddin Ahmed, Shonit Punwani

**Affiliations:** 1 UCL Centre for Medical Imaging, 2nd Floor Charles Bell House, 43 – 45 Foley Street, London, W1W 7TS UK; 2 Medical Physics, University College London Hospital, 235 Euston Road, London, NW1 2BU UK; 3 Department of Urology, University College Hospital, 235 Euston Road, London, NW1 2BU UK; 4 Department of Urology, Imperial College London, Fulham Palace Road, Hammersmith, London, W6 8RF UK

**Keywords:** Magnetic resonance imaging, Prostate, Choline, Positron emission tomography

## Abstract

**Objectives:**

To determine the diagnostic accuracy and interobserver concordance of whole-body (WB)-MRI, vs. ^99m^Tc bone scintigraphy (BS) and ^18^fluoro-ethyl-choline (^18^F-choline) PET/CT for the primary staging of intermediate/high-risk prostate cancer.

**Methods:**

An institutional review board approved prospective cohort study carried out between July 2012 and November 2015, whereby 56 men prospectively underwent 3.0-T multiparametric (mp)-WB-MRI in addition to BS (all patients) ± ^18^F-choline PET/CT (33 patients). MRI comprised pre- and post-contrast modified Dixon (mDixon), T2-weighted (T2W) imaging, and diffusion-weighted imaging (DWI). Patients underwent follow-up mp-WB-MRI at 1 year to derive the reference standard. WB-MRIs were reviewed by two radiologists applying a 6-point scale and a locked sequential read (LSR) paradigm for the suspicion of nodal (N) and metastatic disease (M1a and M1b).

**Results:**

The mean sensitivity/specificity of WB-MRI for N1 disease was 1.00/0.96 respectively, compared with 1.00/0.82 for ^18^F-choline PET/CT. The mean sensitivity and specificity of WB-MRI, ^18^F-choline PET/CT, and BS were 0.90/0.88, 0.80/0.92, and 0.60/1.00 for M1b disease. ROC-AUC did not show statistically significant improvement for each component of the LSR; mean ROC-AUC 0.92, 0.94, and 0.93 (*p* < 0.05) for mDixon + DWI, + T2WI, and + contrast respectively. WB-MRI had an interobserver concordance (*κ*) of 0.79, 0.68, and 0.58 for N1, M1a, and M1b diseases respectively.

**Conclusions:**

WB-MRI provides high levels of diagnostic accuracy for both nodal and metastatic bone disease, with higher levels of sensitivity than BS for metastatic disease, and similar performance to ^18^F-choline PET/CT. T2 and post-contrast mDixon had no significant additive value above a protocol comprising mDixon and DWI alone.

**Key Points:**

*• A whole-body MRI protocol comprising unenhanced mDixon and diffusion-weighted imaging provides high levels of diagnostic accuracy for the primary staging of intermediate- and high-risk prostate cancer.*

*• The diagnostic accuracy of whole-body MRI is much higher than that of bone scintigraphy, as currently recommended for clinical use.*

*• Staging using WB-MRI, rather than bone scintigraphy, could result in better patient stratification and treatment delivery than is currently provided to patients worldwide.*

**Electronic supplementary material:**

The online version of this article (10.1007/s00330-018-5813-4) contains supplementary material, which is available to authorized users.

## Introduction

Since patient survival in intermediate- and high-risk prostate cancer depends heavily on TNM stage [1], accurate tumour staging should underpin all prognostication and management decisions. However, the mainstay of imaging-based staging decisions is still based on ^99m^Tc bone scintigraphy (BS) ± pelvic CT, as is still advised at least eight international guidelines [2]. Whilst these modalities are simple to implement, their diagnostic accuracy remains severely limited [3, 4], which has driven the development of a number of imaging methods for cancer staging,

While choline PET/CT offers improved sensitivity and specificity for both nodal [4, 5] and metastatic disease vs. BS and conventional CT [4], PET involves ionising radiation exposure and has a spatial resolution limited to 5 mm [6], poor contrast resolution, and financial and logistical difficulties which limit its use. Whole-body (WB)-MRI offers potential solutions to these problems, with improved spatial and contrast resolution [7], lack of ionising radiation, and comparable performance characteristics to choline PET/CT as shown by a number of early studies [8–12]. Although prostate-specific membrane antigen (PSMA) PET/CT has demonstrated considerable early promise [13], its availability is limited and incurs considerable cost. Furthermore, since prostate cancer patients commonly undergo multiparametric (mp) prostate MRI, the possibility of a one-stop staging modality has been raised [12] whereby mp-WB approaches could also be applied. However, the interobserver concordance of WB-MRI remains uncertain, as does a definition of what constitutes an optimal acquisition. Further validation regarding diagnostic accuracy is also required.

The primary aim of the present study is to determine the diagnostic accuracy of WB-MRI vs. BS and ^18^fluoro-ethyl-choline (^18^F-choline) PET/CT for the primary staging of intermediate- and high-risk prostate cancer, using a multiparametric vertex-to-feet acquisition protocol and a locked sequential read (LSR) paradigm to determine the additive value of each MRI sequence. Secondary aims include assessment of lesion distribution, interobserver concordance, and intermodality concordance with BS and ^18^F-choline PET/CT. We hypothesise that (i) WB-MRI has a higher diagnostic accuracy than BS, (ii) WB-MRI has good interobserver concordance, and (iii) a multiparametric whole-body acquisition has a greater diagnostic accuracy than T1-weighted imaging plus DWI.

## Materials and methods

**Fig. 1:**
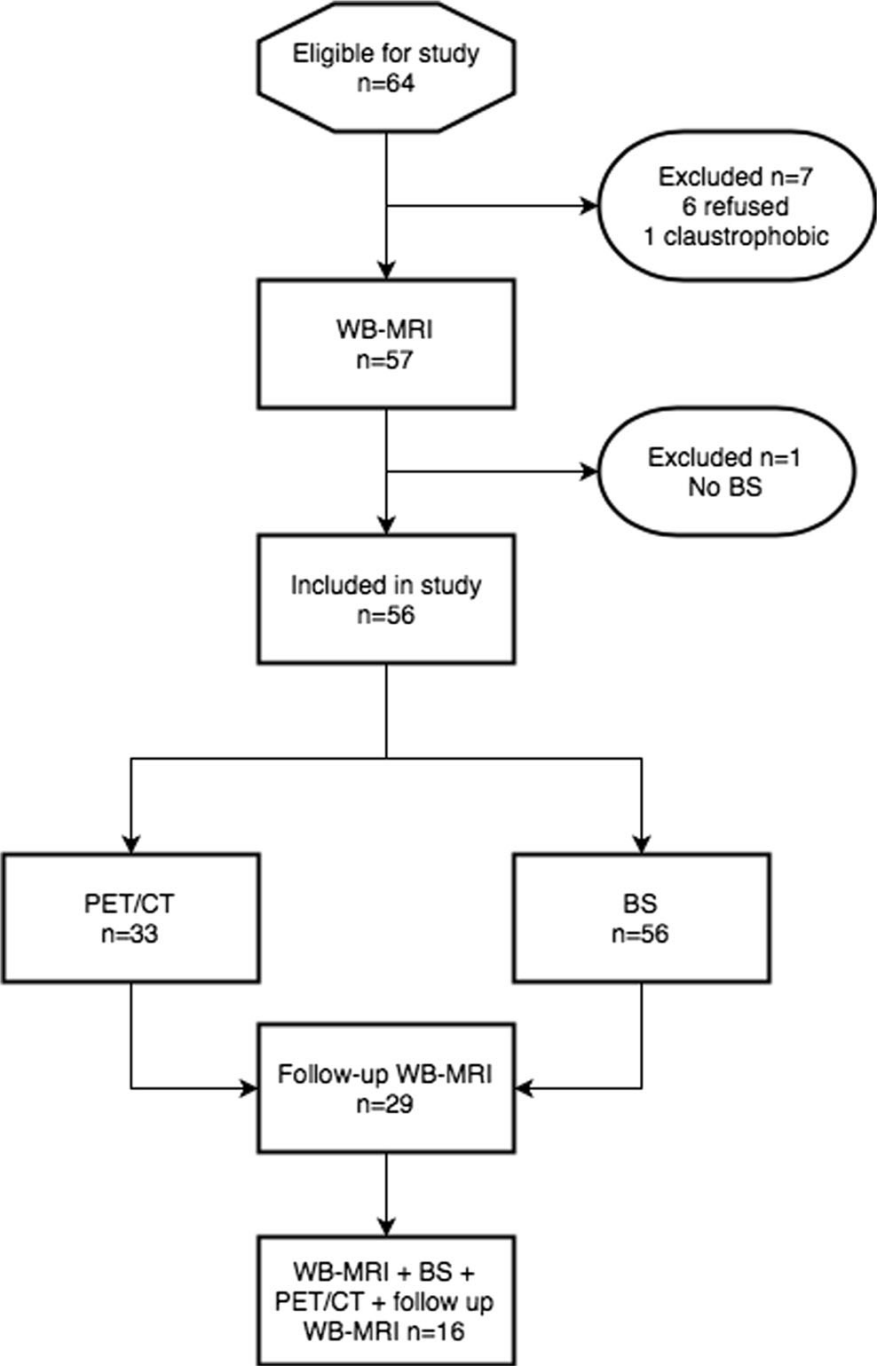
Patient recruitment flow diagram

Our institutional review board approved this prospective single-centre study. Informed written consent was obtained from each participant, whereby 56 consecutive men (mean age 67.9 years, range 51.9–84.4) were identified at Multidisciplinary Tumour Board (MTB) meetings and recruited to the study between July 2012 and November 2015. Inclusion criteria were (i) men aged 18 or over and (ii) new diagnosis of intermediate- or high-risk prostate cancer according to the D’Amico criteria [14]. Exclusion criteria were (i) contraindications to MRI, e.g. severe claustrophobia or MR unsafe device, (ii) prior therapy for prostate cancer, and (iii) men unable to provide informed consent. A recruitment flow diagram is shown in Fig. 1.

Standard imaging comprised BS in all patients ± ^18^F-choline PET/CT, in 33 patients. The decision to perform a ^18^F-choline PET/CT was made on a case-by-case basis whereby the risk of extraprostatic disease was considered to be high at MTB discussion; however, the result of the WB-MRI was blinded to the MTB members, so it did not influence the decision to perform PET/CT. WB-MRI was performed within a mean of 15.9 days (range 0–49) of BS.

### Multiparametric WB-MRI protocol

All patients were imaged on a 3.0-T wide-bore system (Ingenia, Philips), with whole-body coverage from the vertex to feet using a head coil, two anterior surface coils, and table-embedded posterior coils. Coronal pre-contrast modified Dixon (mDixon), axial T2 turbo spin echo (TSE), and axial diffusion-weighted imaging (DWI) with body signal suppression at 4 *b*-values (b0, b100, b300, and b1000) were performed, from which an ADC map was constructed. Post-contrast mDixon imaging was then carried out following a 20 ml injection of intravenous gadoterate meglumine (Dotarem®, Guebert).

Full acquisition parameters are provided in Table 1.

**Table 1:** Whole-body MRI acquisition parameters.

Imaging plane	T2-TSE	mDixon (pre- and post-contrast)	DWI (b0, b100, b300, b1000)
	Transverse	Coronal	Transverse
TE (ms)	80	1.02/1.8	71
TR (ms)	1228	3.0	6371
FOV (mm × mm)	500 × 300	502 × 300	500 × 306
Voxel size (mm × mm)	1 × 1	2.1 × 2.1	4 × 4.2
Number of slices	40	120	40
Slice thickness (mm)	5	5	5
Acquisition matrix	500 × 286	144 × 238	124 × 72
ETL	91	2	39
Acceleration factor (SENSE)	2	2	2.5
Pixel bandwidth (Hz)	537	1992	3369
Scan time (min)	15.2	5.5 × 2	47

*T2-TSE* T2-weighted turbo spin echo, *mDixon* modified Dixon, *DWI* diffusion-weighted imaging, *TE* time of echo, *TR* time of repetition, *FOV* field of view, *ETL* echo train length, *SENSE* sensitivity encoding

### ^99m^Tc scintigraphy protocol

Whole-body imaging was performed in all patients, using anterior and posterior views, 256 × 1024 matrix, and energy window(s) of 140 keV, 2–4 h after a single injection of Tc^99^ m-methylene diphosphonate (MDP).

### ^18^F-choline PET/CT protocol

Thirty-three patients underwent ^18^F-choline PET/CT on an integrated 64-slice scanner (Discovery VCT; GE Healthcare) from the vertex to mid-thigh, 60 min after an intravenous injection of ^18^F-fluoro-ethyl-choline tracer (198–410 MBq; average activity, 327.4 MBq). A low-dose, unenhanced CT scan was initially performed for attenuation correction and image fusion at 120 keV and 10 mA with couch movement 0.8 s and 30 mm per rotation. Whole-body PET emission images were then acquired and reconstructed using the Houndsfield units from the CT to a resolution of 128 × 128 with 5-mm slice thickness.

### Follow-up WB-MRI

Patients were invited to attend a follow-up WB-MRI 1 year after their initial scan using an identical acquisition protocol to inform the reference standard. Twenty-nine of the fifty-six patients attended the 1-year scan, and 16 of these patients had undergone PET-CT at baseline (Fig. 1). Of the 27 who did not attend, two patients died, 16 refused a second attendance, and 9 were lost to follow-up.

### BS and ^18^F-choline PET/CT image review

Nuclear medicine physicians reviewed the BS and ^18^F-choline PET/CT staging studies as part of standard clinical care using GE Advantage workstations. Disease positivity was defined as accumulation of radiotracer, greater than the surrounding background and incompatible with normal physiological activity.

### WB-MRI review

Images were prepared for review using the scanner workstation for mDixon images and Osirix (v. 7.0 Pixmeo). Two board-certified radiologists (reader 1, NR with 12 years of experience and reader 2, HS with 9 years of experience) independently reviewed anonymised WB-MR datasets using an Osirix workstation (v. 7.0 Pixmeo), aware of the presenting serum prostate-specific antigen (PSA) level only and blinded to all other clinical and imaging results.

The body was divided into nine nodal regions (external iliac, internal iliac, common iliac, paraaortic, presacral, other abdominal, inguinal, thoracic, and neck) using standard anatomic definitions. Ten skeletal sites were assessed for the presence of disease (skull, cervical spine, thoracic spine, lumbar spine, pelvis, sternum, clavicle/scapula, ribs, upper limb, and lower limb). Scans were reviewed using a LSR paradigm, whereby each radiologist initially reviewed the unenhanced mDixon and DWI and scored the suspicion of disease at each site using a 1–6 ordinal scale (1, definitely not present; 2, probably not present; 3, possibly not present; 4, possibly present; 5, probably present; 6, definitely present) for each disease site, according to the TNM 7th edition staging system (N0/N1, M1a/M1b/M1c).

The score was specifically assigned at each site using the imaging features as follows on the pre-contrast mDixon and DWI sequences: 1, no lesion evident; 2, poorly visible lesion evident on T1-weighted imaging only—low T1 signal bone focus or lymph node visible but not convincing for malignant involvement or < 5-mm short axis diameter (SAD); 3, definite lesion visible on T1-weighted imaging but not DWI, lymph node 6–9 mm in SAD; 4, definite lesion on T1 with mild increase in high *b*-value diffusion signal vs. background noise, lymph node 10–12 mm in SAD; 5, definite lesion seen on T1 and DWI with moderate increase in high *b*-value DWI signal vs. background noise, lymph node 12–14 mm SAD; 6, definite lesion seen on T1 and DWI with large increase in high *b*-value signal vs. background noise, lymph node ≥ 15 mm SAD. T2W images were then revealed and sites rescored as negative or positive. A negative score was assigned where there was no lesion or where features favoured benignity (e.g. fatty nodal hilum or high T2 signal of haemangioma), and a positive score assigned for features that favour malignancy (rounded nodal morphology, low T2 signal in node or bone lesion). Positive T2 appearances were scored up a point on the initial 1–6 scale (e.g. 3/6 on mDixon/DWI becomes 4/6), and negative T2 appearances were scored down a point (3/6 on mDixon/DWI becomes 2/6). Lastly, post-contrast mDixon images were revealed and a final WB-MRI score was assigned. Here, lesional enhancement was scored up a point on the 1–6 scale and down a point if there was no enhancement. The time to report WB-MRI studies was recorded for both readers. Where discordancy arose between the two radiologists, a third board-certified radiologist with 12 years of experience (reader 3, SP) adjudicated and rescored discordant sites using all available MR images, also aware of the PSA level only.

## Derivation of WB-MRI reference standard

A panel comprising two board-certified radiologists (SP and EJ with 12 and 6 years of experience), and an oncologist with 8 years of experience (RD) reviewed baseline and follow-up WB-MRIs, in combination with all available clinical and radiological information at least 1 year from baseline imaging to carry out a patient-based analysis, and assign patients into the following categories using the definitions below for all modalities. Patients were included in the M1a sensitivity/specificity analysis if:

(i) they had undergone baseline WB-MRI and had a positive ^18^F-choline PET/CT for nodal assessment; or
(ii) they had a baseline WB-MRI and negative ^18^F-choline PET/CT and also underwent follow-up WB-MRI to allow final arbitration.

Similarly, patients were included in the M1b sensitivity/specificity analysis if:

(i) they had undergone baseline WB-MRI and had a positive ^18^F-choline PET/CT or BS for bone assessment; or
(ii) they had a baseline WB-MRI and negative ^18^F-choline PET/CT and BS, and also underwent follow-up WB-MRI to allow final arbitration.

The reference standard was subsequently derived using the following definitions:

- True positive (TP) sites:

(i) Lesion on WB-MRI (defined as suspicion level 4/5/6) which is BS and/or ^18^F-choline PET/CT positive (if performed). Follow-up WB-MRI (if performed) also demonstrates lesion progression without systemic therapy, decrease with systemic therapy, or new lesions. (ii) Lesion on WB-MRI which is negative on conventional imaging but progresses on WB-MRI follow-up without systemic therapy, or new lesions appear on WB-MRI follow-up.

- True negative (TN) sites: No lesion on WB-MRI (defined as suspicion level 1/2/3) and BS and ^18^F-choline PET/CT concordantly negative. In addition, follow-up WB-MRI remains negative. WB-MRI follow-up was therefore required to assign TN cases.
- False positive (FP) sites: Lesion on WB-MRI that was BS and ^18^F-choline PET/CT negative and unchanged at follow-up. WB-MRI follow-up was therefore required to assign FP cases.
- False negative (FN) sites: No lesion on WB-MRI but positive BS and/or ^18^F-choline PET/CT.

**Fig. 2:**
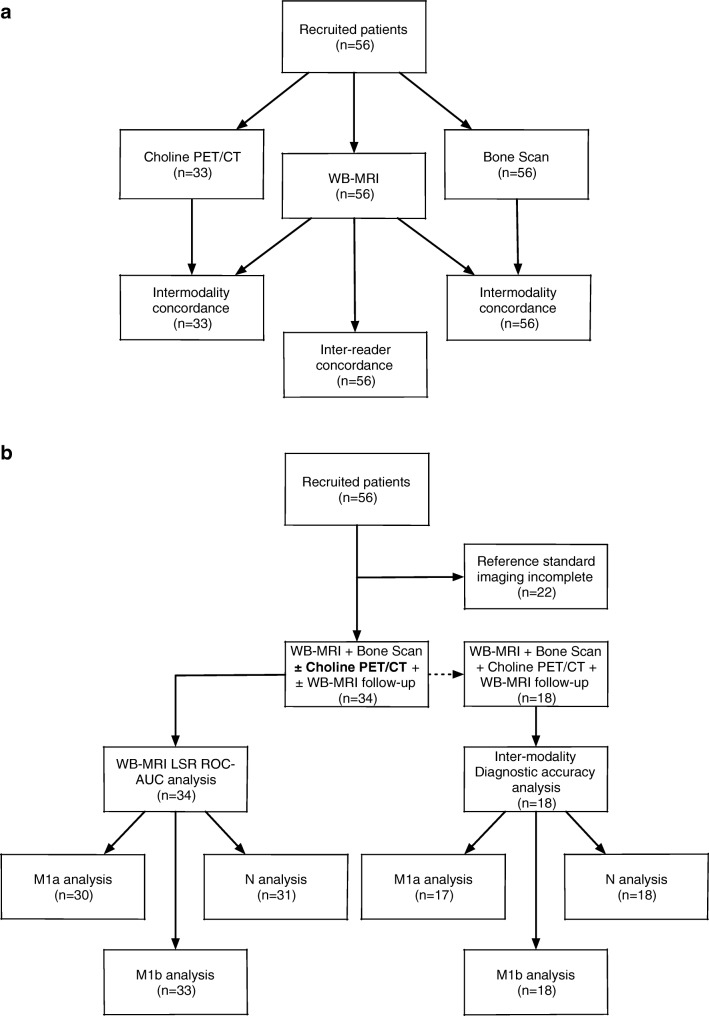

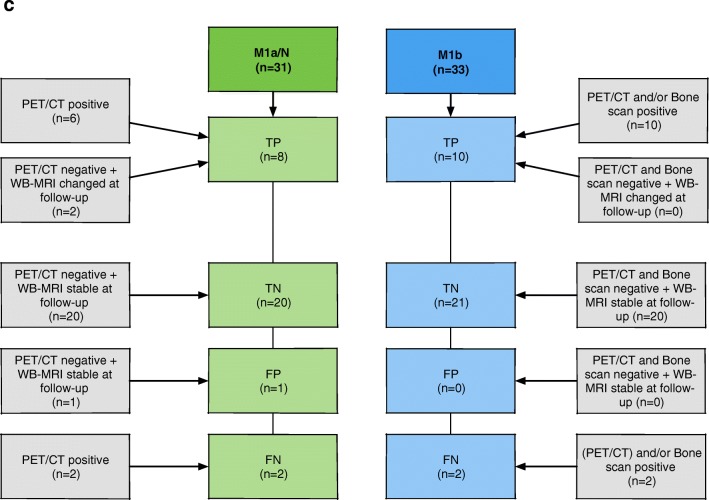
Flow diagrams of the statistical methods used in the study. **a** Flow diagram of intermodality and inter-reader concordance. **b** Flow diagram for WB-MRI LSR ROC-AUC and intermodality diagnostic accuracy analyses. **c** Patient-based reference standard

A flow diagram of the statistical methods and reference standard used in this study is provided in Fig. 2.

A similar reference standard was used to compare BS and ^18^F-choline PET/CT, whereby positive scans concordant with WB-MRI were considered as true positive, with follow-up WB-MRI used for arbitration of discordant and negative BS and ^18^F-choline PET/CT findings. The details for this reference standard are provided in the supplementary materials.

### Statistical analysis

Statistical analysis was performed using SPSS Statistics version 23 (2015, IBM) as below:

1. The distribution of positive lesions for each staging modality (BS, ^18^F-choline PET/CT, and WB-MRI) for local nodal (N0/N1) and metastatic disease (M1a/M1b/M1c) using the TNM classification, following the adjudication of discordant sites by the third board-certified radiologist. Percentages were recorded (i) for all patients (*n* = 56) and (ii) for patients undergoing ^18^F-choline PET/CT (*n* = 33) (Fig. 2a).
2. The inter-reader agreement of WB-MRI (*n* = 56) and agreement between WB-MRI (following the consensus read) and BS (*n* = 56) and ^18^F-choline PET/CT (*n* = 33) following adjudication by the third board-certified radiologist were assessed using Cohen’s *κ* statistics, interpreted according to Landis and Koch [15], whereby < 0 indicates no agreement; 0–0.20, slight; 0.21–0.40, fair; 0.41–0.60, moderate; 0.61–0.80, substantial; and 0.81–1, almost perfect agreement (Fig. 2a).
3. Receiver operator characteristic area under the curve (ROC-AUC) was calculated for WB-MRI studies (Fig. 2b), for both readers following each component of the LSR, applying thresholds for each level of suspicion (1–6) vs. the reference standard (Fig. 2c). Differences in ROC-AUC values for each component of the LSR were assessed according to [16], using a significance level of *p* < 0.05. Youden’s index [17] was used to determine the optimal cutoff of the ROC curve providing the highest combination of sensitivity and specificity.
4. An intermodality diagnostic accuracy study was performed (Fig. 2b), whereby the sensitivity, specificity positive (PPV), and negative (NPV) predictive values were then determined at each TNM stage for a cohort of patients undergoing BS, ^18^F-choline PET/CT, and WB-MRI against the same reference standard (Fig. 2c), using a score of ≥ 4 as positive for the WB-MRI.

## Results

Fifty-six patients (mean age 67.9 years, range 51.9–84.4 years), median PSA 20.05 (IQR 10.07–61.20). Fifty patients were ‘high-risk’ and 6 patients ‘intermediate-risk’. Maximum Gleason score was 3 + 3 for two patients, 3 + 4 for nineteen patients, 4 + 3 for fourteen patients, 4 + 4 for five patients, 4 + 5 for thirteen patients, and 5 + 5 for one patient.

The mean time of radiologists to report each component of the LSR was 15 min for mDixon + DWI, and an additional 6.5 min for T2W and 4 min for post-contrast scans.

No suspicious lesion (scoring 4, 5, or 6) was identified below the mid-thigh level on any imaging modality. Two cases had suspicious lesions in the cervical and thoracic spine; otherwise, no disease was identified above the diaphragm. The review panel also found that all sites of positive disease on both BS, ^18^F-choline PET/CT, and WB-MRI were anatomically matched.

The distribution of N/M disease for each imaging modality (BS, ^18^F-choline PET/CT, and WB-MRI) is presented in Table 2.

**Table 2:** Distribution of lesions on each imaging modality. The first row for each N/M stage represents a comparison between patients who underwent BS, ^18^F-choline PET/CT, and WB-MRI (*n* = 33), and the second row represents a comparison between the patients who underwent BS and WB-MRI (*n* = 56)

	BS	^18^F-choline PET/CT	WB-MRI
N0	–	23/33 (69.7%)	26/33 (78.8%)
	–	–	43/56 (76.8%)
N1	–	10/33 (30.3%)	7/33 (21.2%)
	–	–	13/56 (23.2%)
M0	30/33 (90.9%)	21/33 (63.6%)	22/33 (66.7%)
	43/56 (76.8%)	–	34/56 (60.7%)
M1a	–	6/33 (18.2%)	3/33 (9.1%)
	–	–	6/56 (10.7%)
M1b	3/33 (9.1%)	6/33 (18.2%)	8/33 (24.2%)
	13/56 (23.2%)	–	16/56 (28.6%)
M1c	–	0/33 (0%)	0/33 (0%)
	–	–	0/56 (0%)

Concordance statistics (*κ*) between WB-MRI readers, between WB-MRI consensus and BS, and between WB-MRI consensus and ^18^F-choline PET/CT are presented in Table 3.

**Table 3:** Interobserver and intermodality concordances

	Local nodes (N1)	Metastatic nodes (M1a)	Metastatic bones (M1b)
Interobserver concordance for WB-MRI (*n* = 56)	0.79	0.68	0.58
Concordance of WB-MRI vs. BS (*n* = 56)	–	–	0.68
Concordance of WB-MRI vs. ^18^F-choline PET/CT (*n* = 33)	0.77	0.37	0.64

*WB-MRI* whole-body MRI, *BS*^99m^Tc bone scintigraphy

ROC-AUC statistics for ‘TNM’-based nodal and metastatic status following each part of the LSR are presented in Table 4 against the follow-up based reference standard.

**Table 4:** ROC-AUC for each component of the LSR, performed as part of a patient-based analysis according to the reference standard

ROC-AUC	N0/N1 (*n* = 30)	M1a (*n* = 31)	M1b (*n* = 33)	Mean ROC-AUC
Reader 1				
mDixon + DWI	0.97 (0.91–1.00)	0.99 (0.96–1.00)	0.86 (0.72–1.00)	0.94
+ T2-TSE	0.98 (0.94–1.00)	0.99 (0.95–1.00)	0.93 (0.84–1.00)	0.96
+ contrast	0.98 (0.94–1.00)	0.97 (0.91–1.00)	0.90 (0.76–1.00)	0.95
Reader 2				
mDixon + DWI	0.94 (0.81–1.00)	0.87 (0.60–1.00)	0.86 (0.73–1.00)	0.89
+ T2-TSE	0.94 (0.82–1.00)	0.87 (0.60–1.00)	0.94 (0.83–1.00)	0.91
+ contrast	0.94 (0.82–1.00)	0.87 (0.60–1.00)	0.93 (0.82–1.00)	0.91

*ROC-AUC* receiver operator characteristic area under the curve, *LSR* locked sequential read, *TSE* turbo spin echo. Figures in parentheses represent 95% confidence intervals

No significant differences were detected between the mean ROC-AUC for each component of the LSR (*p* < 0.05), so the simplest WB-MRI combination was chosen for further analysis (DWI + mDixon). Youden’s index confirmed the optimal cutoff of the ROC-AUC was ≥ 4 in all cases. The sensitivity and specificity for BS, ^18^F-choline PET/CT, and WB-MRI were therefore calculated using a threshold of ≥ 4 as positive against the follow-up reference standard. Results are displayed in Table 5, along with their numerators and denominators.

**Table 5:** Performance characteristics of BS, ^18^F-choline PET/CT, and WB-MRI carried out as a patient-based analysis vs. the reference standard

	N1 (*n* = 18)	M1a (*n* = 17)	M1b (*n* = 18)
BS			
Sensitivity	–	–	0.60 (3/5)
Specificity			1.00 (13/13)
PPV			1.00 (3/3)
NPV			0.87 (13/15)
^18^F-choline PET/CT			
Sensitivity	1.00 (7/7)	0.75 (3/4)	0.80 (4/5)
Specificity	0.82 (9/11)	0.92 (12/13)	0.92 (12/13)
PPV	0.77 (7/9)	0.75 (3/4)	0.80 (4/5)
NPV	1.00 (9/9)	0.92 (12/13)	0.92 (12/13)
WB-MRI: reader 1			
Sensitivity	1.00 (7/7)	1.00 (4/4)	0.80 (4/5)
Specificity	0.91 (10/11)	0.85 (11/13)	1.00 (13/13)
PPV	0.88 (7/8)	0.67 (4/6)	1.00 (4/4)
NPV	1.00 (10/10)	1.00 (11/11)	0.93 (13/14)
WB-MRI: reader 2			
Sensitivity	1.00 (7/7)	0.50 (2/4)	1.00 (5/5)
Specificity	1.00 (11/11)	1.00 (13/13)	0.76 (10/13)
PPV	1.00 (7/7)	1.00 (2/2)	0.62 (5/8)
NPV	1.00 (11/11)	0.86 (13/2)	1.00 (10/10)
WB-MRI mean			
Sensitivity	1.00	0.75	0.90
Specificity	0.96	0.93	0.88
PPV	0.94	0.83	0.81
NPV	1.00	0.93	0.97

*WB-MRI* whole-body MRI, *BS*^99m^Tc bone scintigraphy, *NPV* negative predictive value, *PPV* positive predictive value. WB-MRI mean is the mean of the two readers

**Fig. 3:**
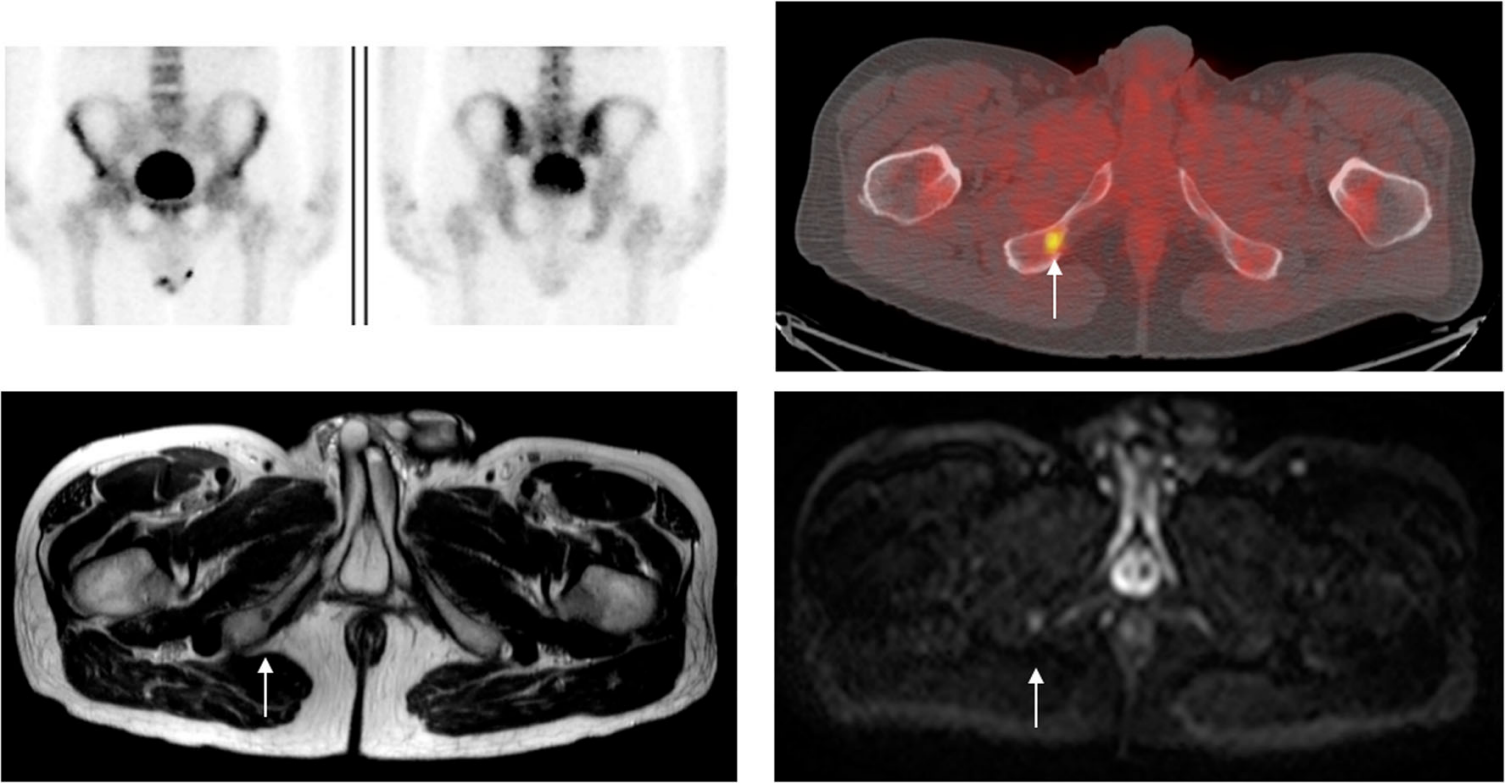
Example of discordant skeletal site in a 64-year-old man with a serum prostate-specific antigen level of 25.2. Top left: Negative ^99m^Tc bone scintigram (BS), Top right: ^18^F-choline PET/CT showing an area of increased tracer avidity at the right inferior pubic ramus consistent with a metastasis. Bottom left: Axial T2W TSE showing the lesion is of low signal intensity. Bottom right: the lesion has increased diffusion-weighted signal on *b* = 1000s/mm^2^. The lesion was considered as an example of a true positive whole-body MRI, true positive ^18^F-choline PET/CT, and false negative BS

**Fig. 4:**
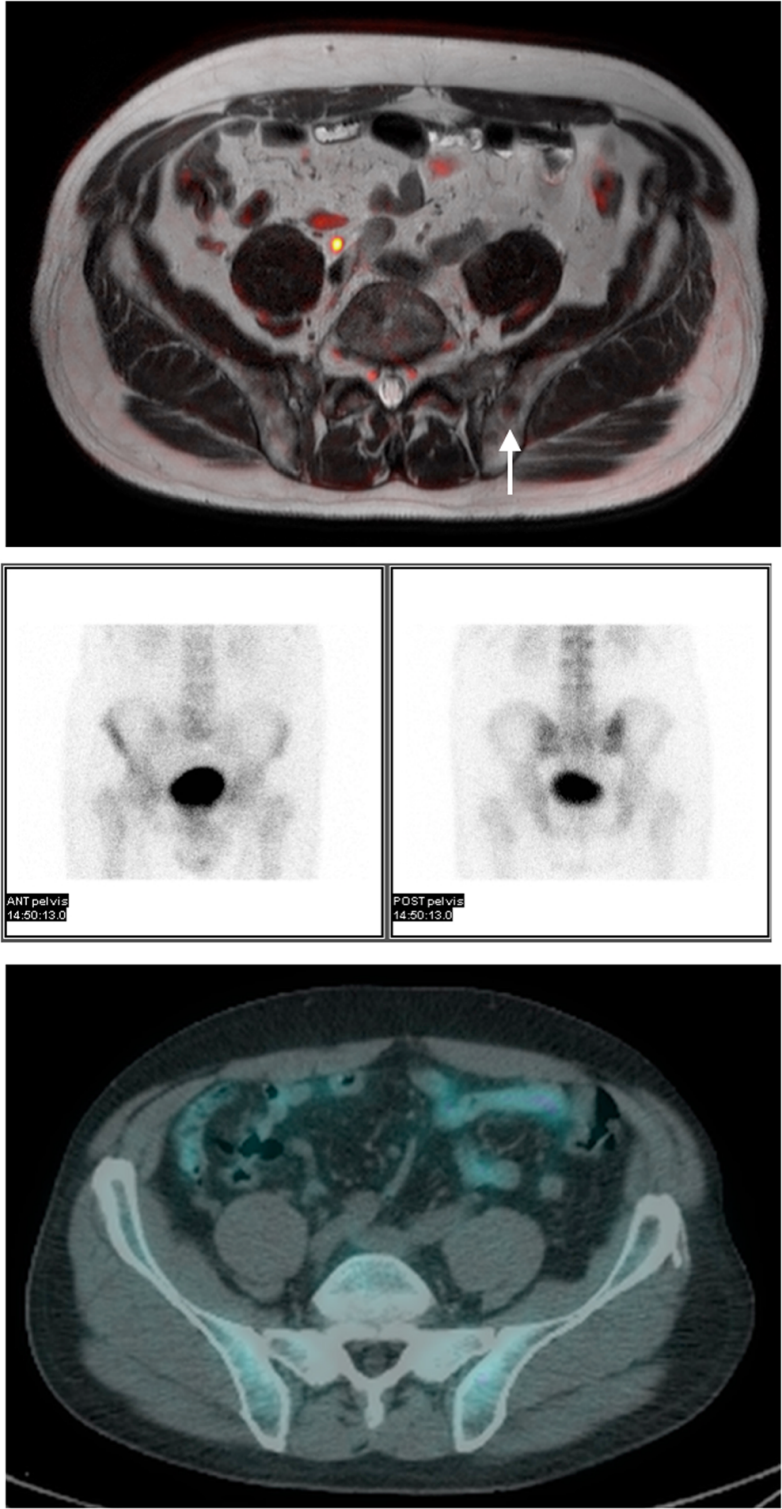
Example of a discordant skeletal site in a 73-year-old man with a serum prostate-specific antigen level of 19.4. Top: Axial T2-weighted turbo spin echo with fused *b* = 1000s/mm^2^ showing a metastasis in the left iliac body, which is occult on BS (middle) and ^18^F-choline PET/CT (bottom). The site was considered to represent a true positive whole-body MRI, false negative ^18^F-choline PET/CT, and false negative ^99m^Tc bone scintigram

Typical examples of lesions missed on BS which are detected by WB-MRI are provided in Figs. 3 and 4.

## Discussion

WB-MRI is gaining momentum as a staging modality in prostate cancer, but requires further validation prior to being introduced into clinical practice. Firstly, our results show that WB-MRI detected more positive bony metastatic (M1b) disease than ^18^F-choline PET/CT and BS with 8, 6, and 3 positive lesions for WB-MRI, ^18^F-choline PET/CT, and BS respectively. We then confirmed that WB-MRI had the highest sensitivity of all modalities for detecting metastatic bone disease: 0.90 vs. 0.80 for ^18^F-choline PET/CT and 0.60 for BS for specificities of 0.88, 0.92, and 1.00 respectively. This finding is in accordance with a meta-analysis which compared the diagnostic accuracy of BS, ^18^F-choline PET/CT, and WB-MRI and gave pooled sensitivities and specificities of 0.97/0.95, 0.91/0.99, and 0.79/0.82 for WB-MRI, ^18^F-choline PET/CT, and BS [4] respectively. High diagnostic sensitivity could reflect the fact that DWI sequences are designed to probe small changes in tissue microstructure, as found in the early cellular phase of a metastasis, before a sclerotic reaction has been effected in bone [18].

High and very similar sensitivities/specificities were also shown for WB-MRI and ^18^F-choline PET/CT for nodal disease, with values of 1.00/0.96 and 1.00/0.82 for N1 disease and 0.75/0.93 and 0.75/0.92 for M1a disease respectively. Both of the cross-sectional modalities therefore appear more accurate than conventional CT, which is again in accordance with a meta-analysis which reported pooled sensitivities and specificities of 0.42/0.82 for CT [3], vs. 0.49/0.95 for choline PET/CT [5]. MRI studies which incorporate DWI into their scanning protocols report a heterogenous sensitivity for lymph nodes which ranges from 0.17 [19] to 0.73 [20]*.* Whilst both of these studies used extended pelvic lymph node dissection as the reference standard, the lower sensitivity reported by Pinaquy and colleagues [19] could relate to their chosen *b*-values of 0 and 100 s/mm^2^, which contravenes the recommendations of international consensus guidelines [21], and emphasises the need for optimised scanning technique. In concordance with the findings of our study, the specificity of MRI for nodal detection is thought to be high, with a limited number of studies quoting values ranging from 86% [20] to 98% [22].

A potential strength of our study was the use of a vertex-to-feet protocol which enabled direct comparison with BS and could assess potential lesions outside of the field of view for ^18^F-choline PET/CT. Whole-body cross-sectional studies regarding disease distribution in the PSA screening era are welcome since strongest data regarding disease distribution is provided by an autopsy study prior to PSA screening era, which did not routinely examine the peripheral skeleton [23]. Complete body coverage has been both suggested [24, 25] and deemed unnecessary [9], which is perhaps could partially be due to the uncertainty regarding disease distribution in the PSA screening era. Since no lesions were detected below the knee or extravertebral lesions above the diaphragm, our data suggests that scanning below the knee may indeed be unnecessary, and a cervical and thoracic spine MRI may be a reasonable compromise for detecting disease above the diaphragm, and is in keeping with the findings of another study [9], which reported all patients with peripheral metastases occurring in high-risk prostate cancer (60 in total) also had vertebral metastases, and no metastases occurred below the knee. With further confirmatory work, scanning the abdomen, pelvis, and femora using pre-contrast mDixon and DWI at 2 *b*-values paired with a whole spine MRI as a routine staging examination could be applied and would have approximately 700 images, vs. 12,000 images per patient in the present study. Reducing the number of images may further improve the interobserver concordance by reducing the complexity of imaging datasets. We found interobserver concordance to be ‘substantial’ for N1 and M1a disease (*κ* = 0.79 and 0.68 respectively), and ‘moderate’ for M1b (0.58), whereby the lower concordance in bone metastases could be explained by the non-specific features of bone lesions on MRI, and the fact that acquisitions were tailored for WB cancer staging rather than bone lesion characterisation. Furthermore, the more subjective criteria applied for assessing bone lesions vs. nodal size measurements may have given rise to further heterogeneity in the data and thus lower levels of concordance.

The LSR paradigm allowed the incremental value of additional sequences to be assessed, whereby adding T2W and post-contrast mDixon sequences did not improve ROC-AUC significantly. These results could be used to streamline WB-MRI scanning protocols in research and clinical practice. For example, performing pre-contrast mDixon + DWI alone could save 10-min reporting time and 20-min scan time and avoid the need for cannulation and gadolinium administration. Furthermore, as suggested by the MET-RADS-P consensus guidelines [25], the use of 2 *b*-values rather than 4 could be sufficient—especially for primary staging purposes, which would reduce scan time by a further 25 min. Whilst the MET-RADS-P guidelines were based on expert opinion, WB-Dixon and DWI were recommended in combination with whole spine T1 and short tau inversion recovery (STIR), meaning our findings provide evidence to support a similar simple scanning protocol when characterising oncological burden in prostate cancer.

Another potential strength of our study was the choice of a reference standard based on follow-up MRI rather than on best value comparator (BVC) alternatives [8] which rely upon imaging tests such as BS and plain radiographs with limited performance characteristics. Whilst TP was assigned without follow-up imaging when BS and MRI were concordant due to the high specificity of BS in the context of prostate cancer, we did not assign TN without MRI follow-up, since genuine lack of sensitivity, i.e. FN results on both modalities, is also possible.

The limitations of this study include patient number, its single-centre nature, and a relatively low number of positive cases. Furthermore, not all patients underwent ^18^F-choline PET/CT, meaning patients with a negative BS in whom the suspicion for metastatic disease remained high may have been more likely to be selected for ^18^F-choline PET/CT vs. patients with clear evidence of metastases on BS, leading to lower apparent levels of diagnostic accuracy for ^18^F-choline PET/CT.

In addition, not all patients underwent WB-MRI follow-up at 1 year, which could lead to selection bias, e.g. patients who were feeling well or unwell at the time of follow-up may be more likely to refuse the second scan. However, the most common reason provided at telephone consultation was that they had incurred too many imaging tests and therefore declined further participation. Whilst incorporation bias likely gave rise to the high values of sensitivity and specificity (e.g. vs. pelvic lymph node dissection (PLND) as a nodal reference standard), it would not have been practical or ethically acceptable to perform nodal dissection for the purposes of the study, and selecting patients who are undergoing PLND would incur spectrum bias.

Further work could include performing a lesion-based analysis in the same cohort of patients, whereby the number of lesions detected and their anatomical sites could be established. Further validation of WB-MRI could also be carried out in multicentre trials, where economic and clinical utility could also be considered. The findings of the present study are not limited to WB-MRI and can be used to inform rational PET-MRI protocols, e.g. in combination with prostate-specific membrane antigen (PSMA) PET tracer.

## Conclusion

WB-MRI provides high levels of diagnostic accuracy for both nodal and metastatic bone disease, with higher levels of sensitivity than BS for metastatic disease, and similar performance to ^18^F-choline PET/CT. T2 and post-contrast mDixon had no significant additive value above a protocol comprising mDixon and DWI alone.

## Electronic supplementary material

ESM 1 (PDF 51 kb)

## Abbreviations

BS: Bone scintigraphy
BVC: Best value comparator
DWI: Diffusion-weighted imaging
LSR: Locked sequential read
mp-WB-MRI: Multiparametric whole-body MRI
MTB: Multidisciplinary tumour board
PLND: Pelvic lymph node dissection
PSMA: Prostate-specific membrane antigen
ROC-AUC: Receiver operator characteristic area under the curve
SAD: Short-axis diameter
