# Supplementary figures and images for: Multiparametric whole-body 3.0-T MRI in newly diagnosed intermediate- and high-risk prostate cancer: diagnostic accuracy and interobserver agreement for nodal and metastatic staging
Source: Eur Radiol. 2018 Dec 5;29(6):3159–69. doi: 10.1007/s00330-018-5813-4 (PMC6510859; doi:10.1007/s00330-018-5813-4)

PET/CT

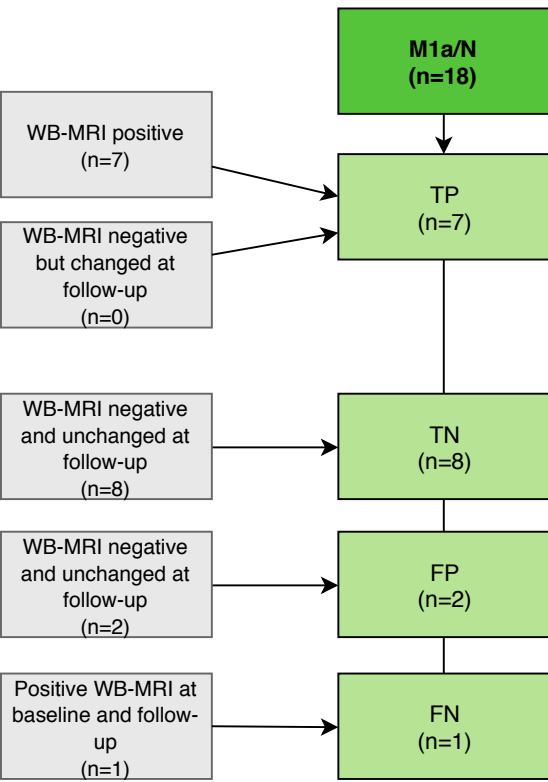

Bone Scan

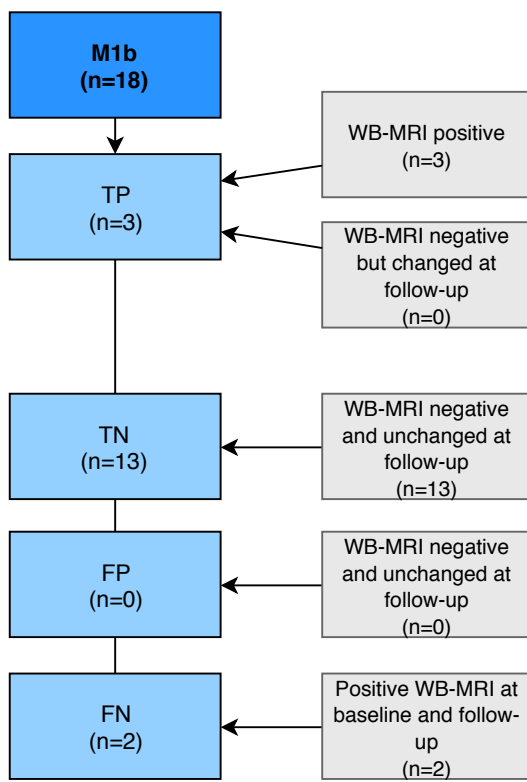

PET/CT

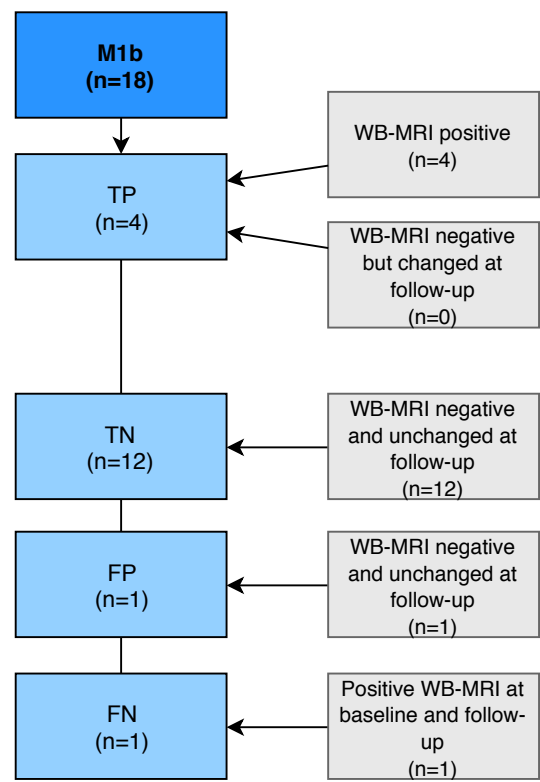

Supplement: Supplementary file 1 — (PDF 51 kb) [file 330_2018_5813_MOESM1_ESM.pdf]
